# Mobile Apps Leveraged in the COVID-19 Pandemic in East and South-East Asia: Review and Content Analysis

**DOI:** 10.2196/32093

**Published:** 2021-11-11

**Authors:** Bohee Lee, Siti Aishah Ibrahim, Tiying Zhang

**Affiliations:** 1 Centre for Population Health Sciences, Usher Institute University of Edinburgh Edinburgh United Kingdom; 2 Faculty of Epidemiology and Population Health London School of Hygiene and Tropical Medicine London United Kingdom

**Keywords:** mobile apps, applications, eHealth, mHealth, mobile health, digital health, telemedicine, telehealth, COVID-19, coronavirus, pandemic, public health, health policy

## Abstract

**Background:**

The COVID-19 pandemic increased attention to digital tools to support governmental public health policies in East and South-East Asia. Mobile apps related to the COVID-19 pandemic continue to emerge and evolve with a wide variety of characteristics and functions. However, there is a paucity of studies evaluating such apps in this region, with most of the available studies conducted in the early days of the pandemic.

**Objective:**

This study aimed to examine free apps developed or supported by governments in the East and South-East Asian region and highlight their key characteristics and functions. We also sought to interpret how the release dates of these apps were related to the commencement dates of other COVID-19 public health policies.

**Methods:**

We systematically searched for apps in Apple App Store and Google Play Store and analyzed the contents of eligible apps. Mobile apps released or updated with COVID-19–related functions between March 1 and May 7, 2021, in Singapore, Taiwan, South Korea, China (mainland), Japan, Thailand, Hong Kong, Vietnam, Malaysia, Indonesia, and the Philippines were included. The CoronaNet Research Project database was also examined to determine the timeline of public health policy commencement dates in relation to the release dates of the included apps. We assessed each app’s official website, media reports, and literature through content analysis. Descriptive statistics were used to summarize relevant information gathered from the mobile apps using RStudio.

**Results:**

Of the 1943 mobile apps initially identified, 46 were eligible, with almost 70% of the apps being intended for the general public. Most apps were from Vietnam (n=9, 20%), followed by Malaysia, Singapore, and Thailand (n=6 each, 13%). Of note, most apps for quarantine monitoring (n=6, 13%) were mandatory for the target users or a population subset. The most common function was health monitoring (32/46, 70%), followed by raising public health awareness (19/46, 41%) through education and information dissemination. Other functions included monitoring quarantine (12/46, 26%), providing health resources (12/46, 26%). COVID-19 vaccination management functions began to appear in parallel with vaccine rollout (7/46, 15%). Regarding the timing of the introduction of mobile solutions, the majority of mobile apps emerged close to the commencement dates of other public health policies in the early stages of the pandemic between March and April 2020.

**Conclusions:**

In East and South-East Asia, most governments used mobile health apps as adjuncts to public health measures for tracking COVID-19 cases and delivering credible information. In addition, these apps have evolved by expanding their functions for COVID-19 vaccination.

## Introduction

The role of digital technology has reached new heights, with 93% of the world’s population having access to mobile broadband networks in 2020 [1]. Today, with more than half of the world’s population (approximately 3.8 billion individuals) owning a smartphone, there is enormous potential and increasing opportunity to cost-effectively incorporate mobile apps into pandemic control strategies [2]. Mobile technologies in public health (mHealth), allow individuals to connect with health services, including surveillance, remote monitoring, and health information [3].

mHealth interventions have been continuously evolving in various settings, including resource-limited settings with the surging penetration of smartphones and continuous advancement of relevant technological capabilities [4]. Evidence has shown that mHealth has been used to enable health care providers to reach out to vulnerable individuals, conduct surveillance, and provide treatment, health-related education, and counseling [4-7].

The capabilities of mHealth interventions have grown quickly during the COVID-19 pandemic, but their abundant potential has been constantly predicted by many researchers, even before the pandemic [8]. For instance, a pilot study by Pant Pai et al [9] observed that an unsupervised HIV self-testing strategy using an internet-based mobile app leads to counseling and treatment among patients testing positive in South Africa. A case study in Uganda also highlighted the feasibility of mHealth approaches to implement antimalaria strategies in a transitional country [10].

Since the World Health Organization (WHO) declared COVID-19 a global pandemic in March 2020, the demand for digital tools to reinforce public health measures has drastically increased worldwide [11]. mHealth solutions have been used for early detection, fast screening, patient monitoring, information sharing, education, and treatment management in response to the COVID-19 outbreak [8]. The pandemic has witnessed a rapid proliferation in the application of digital technologies for public health, with many governments around the globe developing mobile apps to reduce the transmission of SARS-CoV-2 [12,13].

Before the advent of COVID-19 vaccines, many governments in East and South-East Asia have gained unprecedented attention for their effective COVID-19 containment and incredibly low death tolls compared to countries in the West [14]. Governments in this region had experienced the consequences of outbreaks such as severe acute respiratory syndrome (SARS) and Middle East respiratory syndrome (MERS). Therefore, they ensured that their public health systems were better prepared for similar outbreaks by establishing early warning systems and relevant policies [15-18]. Critical medical capacities were augmented while early warning systems and relevant policies were established long before COVID-19 was identified [16,18]. In addition, they actively capitalized on technological solutions to contain the pandemic by leveraging existing regional digital infrastructure through the ASEAN Smart Cities Network (ASCN), a collaborative platform working toward a common goal of smart and sustainable urban development [12,19,20]. These experiences also created a culture of mask-wearing, solidarity, and collective responsibility in the general public [21].

Although a number of systematic reviews had looked at COVID-19–related apps available on a global scale, there is a paucity of studies focusing on mobile apps in this region, which share similar cultural characteristics [13,22,23]. Ming et al [24] found that most apps developed in the United States before May 2020 could trace or map COVID-19 cases and had surveillance features but not educational contents. A recent review by Alanzi [13] examined the functionalities of mobile apps developed by governments in 6 countries including Saudi Arabia, Italy, Singapore, the United Kingdom, the United States, and India as of August 2020. Alanzi [13] found that the most prevalent function was contract tracing, while very few apps had functions for raising public awareness and providing COVID-19–related information. Almalki and Giannicchi [25] assessed mobile apps in a total of 51 countries as of September 2020. They demonstrated that the most common function was basic health information followed by contact tracing, self-assessment, live statistics and the latest news [25]. However, only 5 East and South-East Asian countries (Vietnam, Malaysia, Thailand, Singapore, and South Korea) were included in this assessment.

Given the diverse economic sizes and varying digital adaptation in the East and South-East Asian region [26], it is crucial to know how these governments have developed readiness and abilities to deploy digital technologies integrated with public health measures [14]. In addition, considering the evolving nature of the pandemic, there is a need to examine how COVID-19–related mobile apps are used in the public health context, particularly focusing on this region. Therefore, our review aimed to explore COVID-19–related mobile apps that governments in East and South-East Asia have introduced.

## Methods

### Search Strategy

This study adopted a systematic search strategy using a modified version of the PRISMA-ScR (Preferred Reporting Items for Systematic Reviews and Meta-Analyses Extension for Scoping Reviews) guidelines to identify COVID-19–related apps currently freely available in this region and their characteristics and functions [27]. Adjustments were needed because of the different search nature of mobile app stores.

We referred to Bloomberg’s Covid Resilience Ranking evaluating the 53 largest economies on their success at containing the virus (March 2021) [28]. This ranking covers a wide range of COVID-19 statuses, from mortality rates and COVID-19 testing to vaccination and lockdown severity, and quality of life during the pandemic [28]. This ranking involved 11 governments in East and South-East Asia as of March 2021: Singapore, Taiwan, Hong Kong, South Korea, China (mainland), Japan, Thailand, Vietnam, Malaysia, the Philippines, and Indonesia. The summarized details of the scores of each selected government based on Bloomberg’s Covid Resilience Ranking in March 2021 are presented in Multimedia Appendix 1.

The 2 largest app stores worldwide, iOS-based Apple App Store and Android-based Google Play Store, were searched for potentially relevant mobile apps released or updated from March 1 to May 7, 2021. The following search terms were used: “COVID-19,” “COVID,” “coronavirus,” “corona virus,” “corona,” and “SARS-CoV-2.” To circumvent the regional restriction setting for searching apps, we utilized a website, fnd.ios, to look for apps on Apple App Store and changed the region settings in Google Play Store [29,30]. News articles and media reports were also searched to find further eligible apps that may have been missed. For searching the literature, MEDLINE and Google Scholars were explored by combining 2 search strings, including terms related to mobile apps and COVID-19 such as (“digital health” OR “m-health” OR “mobile health” OR “e-health” OR “mobile apps”) AND (“COVID-19” OR “coronavirus” OR “SARS-CoV-2”). Draft searches were piloted in each database and then finalized. Searches were conducted on May 7, 2021, by 2 reviewers (BL and TZ). To identify and examine the mobile app described in the native language (non-English) of the corresponding government, we searched the app’s official website and news reports to determine whether there was any information provided in English. Google Translator was used if the information about the app was unavailable in either English or the 4 languages spoken by the 3 reviewers (Chinese, Korean, Malaysian, and Japanese).

To evaluate when mobile apps were introduced in relation to other public health policies, we utilized the data set of the CoronaNet Research Project collating governmental public health policies worldwide in the context of COVID-19 [31]. This project comprises a data set providing comprehensive government policies across 195 countries, apprehending 18 broad policy types, including timings of each policy. Any ambiguity was resolved through discussion with a reviewer in the CoronaNet Research Project (CC). We selected national-level policies of 11 governments and validated relevant policies by checking data sources. We narrowed 18 policy types to 6, which were deemed to be associated with the functions of mobile apps such as public awareness measure, COVID-19 testing, quarantine monitoring, health monitoring, vaccination, and health resources [31].

### Eligibility Assessment and Selection of Apps

After initial deduplication, 2 authors (SAI, BL) with backgrounds in public health screened mobile apps on the basis of the identified apps’ titles, keywords, and descriptions. Irrelevant apps were excluded during the preliminary screening step. After screening, the 2 reviewers independently assessed the eligibility of mobile apps on the basis of the eligibility criteria. We included apps if they were (1) related to COVID-19, (2) available free of cost with no in-app purchase requirement, (3) released or updated with COVID-19–related functions during the research period, (4) still available to users on the specified search date, (5) developed or supported by governments or authorities, and (6) with full information regarding the app accessible. However, we excluded mobile apps developed by global organizations, nongovernmental organizations, or communities not representing a government or broader regions. Discrepancies were resolved through discussion between 2 reviewers or arbitration by a third reviewer to reach a consensus.

### Data Extraction and Synthesis

We used a modified framework of previous studies and the CoronaNet Project database for data extraction [13,31]. This framework covers key characteristics and functions of mobile apps in accordance with coding and policy definitions by the CoronaNet Research Project [31]. Key characteristics include the country of origin, platform availability (Apple App Store and Google Play Store), release date, developer, target users, uptake requirement, and required technology. Key functions were merged into 6 policy types. Definitions of key functions and lists of subordinate functions are described in Textbox 1.

Based on this framework, we developed a data extraction form, and 2 independent reviewers extracted the relevant data. Each app’s official website, relevant media reports, and literature were assessed through content analysis [32]. Through this technique, we identified and quantified relevant keywords indicating key characteristics and functions [32]. At each step, disagreements were resolved by consensus. In case of persistent disagreement, arbitration by the third reviewer settled the discrepancy. Descriptive statistics were used to summarize relevant information gathered from the mobile apps, using RStudio (version 1.3.1056).

**Definitions of key functions and list of subordinate functions of eligible mobile apps.**

**Public Awareness Measures:** Government efforts to disseminate or gather reliable information about COVID-19

- News or government measures
- Up-to-date statistics
- COVID-19 health information
- Health management guidelines
- COVID-19 related services information
- Hotspot/risk area identification

**COVID-19 Testing:** Government policies to detect COVID-19 cases

- Obtain COVID-19 test
- Report of test results

**Quarantine Monitoring:** Targets of the policy are obliged to isolate themselves for at least 14 days because there is reason to suspect a person is infected with COVID-19

- Regular health check
- Location tracking

**Health Monitoring:** Government policies to monitor the health of individuals to limit the spread of COVID-19

- Digital contact tracing
- Digital check-in
- Alert contacts of COVID-19 cases
- Report suspected cases/rule infringement
- Health code/status generator
- Health/travel declaration
- Self-symptom assessment

**Vaccination:** Government policy made with regards to either the research and development, regulation, production, purchase and distribution of a given COVID-19 vaccine

- Vaccination information
- Vaccination registration/appointment
- Vaccination certificate
- Reporting adverse reactions

**Health Resources:** Government policies that affect the material (eg, medical equipment, number of hospitals for public health) or human (eg, doctors, nurses) health resources of a country

- Virtual medical consultation
- Emergency helpline
- Accessing medical records
- Personal protective equipment distribution

## Results

### Selected Apps

**Figure 1:**
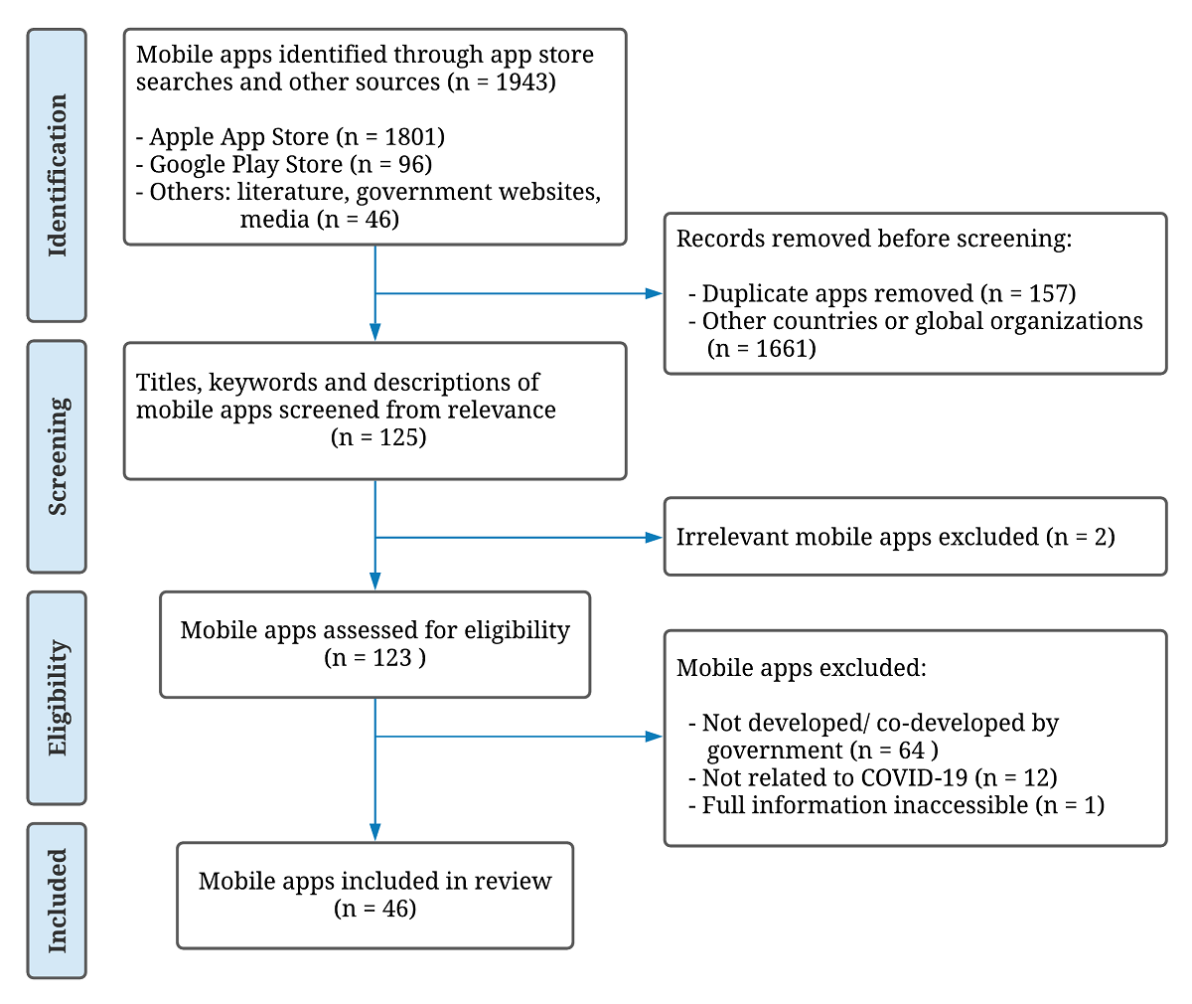
PRISMA (Preferred Reporting Items for Systematic Reviews and Meta-Analyses) flowchart of the search process.

Figure 1 illustrates an overview of the process involved in selecting the apps for study synthesis. A total of 1943 potential apps were obtained through systematic searches, of which 46 met our eligibility criteria. Although 3 of the apps, namely Alipay, WeChat, and My Health Bank, have pre-existed before March 2020, we included them in the review as they have since been updated to include COVID-19–related services during the pandemic.

### Characteristics of the Included Apps

All the included apps were free for users to download and use without any in-app purchase requirements. Furthermore, they were official apps developed or supported by the government and maintained by the relevant authority for COVID-19–related service provision. Descriptive analytics related to the characteristics of the apps were summarized and presented in Table 1. Most of the apps (n=9, 20%) were from Vietnam, followed by Malaysia, Singapore, and Thailand (n=6 apps each, 13%). Almost 98% of the apps were available on both iOS and Android platforms through the Apple App Store and Google Play Store.

In total, 24 (52%) apps were mandatory, with a mandate for target users to install them on their smartphones. Target users were mainly a subset of the population only, for example, people living in high-risk areas with stringent pandemic restrictions and confirmed or suspected COVID-19 cases.

Most of these apps (n=32, 70%) were intended for the general public. Six (13%) apps were especially intended for quarantined people: 4 (9%) apps for quarantined residents and 2 (4%) apps for quarantined inbound travelers. Six (13%) apps targeted travelers: domestic and international travelers (n=2, 4%), international travelers including those who required quarantine (n=3, 7%), and outbound travelers (n=1, 2%). Overall, the GPS was the most required technology (n=28, 61%), followed by Bluetooth (n=16, 35%) and the QR scanner (n=16, 35%). Artificial intelligence (AI), the application programming interface (API), and facial-recognition technology were also utilized in 3 (7%) apps. Details of apps with associated characteristics currently available across 11 governments included in this review are described in Multimedia Appendix 2.

**Table 1:** Overview of the included apps (N=46).

Characteristics		Apps, n (%)
**Origin**		
	China (mainland)	2 (4)
	Hong Kong	3 (7)
	Indonesia	3 (7)
	Japan	4 (9)
	Malaysia	6 (13)
	Philippines	1 (2.2)
	Singapore	6 (13)
	South Korea	3 (7)
	Taiwan	3 (7)
	Thailand	6 (13)
	Vietnam	9 (20)
**Platform**		
	iOS (App Store)	45 (98)
	Android (Google Play Store)	46 (100)
**Uptake requirement**		
	Mandatory	24 (52)
	Voluntary	22 (48)
**Target users**		
	General public	32 (70)
	Travelers: domestic and international	2 (4)
	Travelers: international	1 (2)
	Travelers: requiring quarantine	2 (4)
	Travelers: outbound	1 (2)
	Foreign workers	1 (2)
	Quarantined individuals	4 (9)
	Business owners	1 (2)
	Vaccinated individuals	2 (4)
**Required technology**		
	GPS	28 (61)
	Bluetooth	16 (35)
	QR scanner	16 (35)
	Others^a^	3 (7)

^a^Other technologies include artificial intelligence (n=1), the application programming interface (API) (n=1), and facial recognition (n=1).

### Functions of the Included Apps

Overall, 25 common functions were identified, and they were subsequently organized into 6 overarching domains that characterized the functions of these apps, as shown in Table 2. The functions supported by each app are detailed in Multimedia Appendix 3.

The most common function served by the apps was health monitoring (n=32, 70%). Eleven apps (24%) were used for digital contact tracing by tracking, documenting, and retaining mobile phone users’ encounters with other devices using Bluetooth or GPS technologies. Twelve (26%) apps had the function of alerting the contacts of COVID-19 cases. If one of the app users contracted COVID-19, authorities with access to the data could request the infected user to upload the relevant anonymized data for analysis so that others with the same installed app who were in close contact may be alerted for further action. Eleven apps (24%) served the digital check-in function with the same goal for contact tracing: maintaining an efficient digital log of visitors so that officials could quickly reach out to those who might have been in close contact with a COVID-19 case present in the same events or premises.

The second-most common function associated with the apps was public health awareness (n=19, 41%). More than half of these apps were developed to disseminate the latest news (n=12, 26%) and up-to-date statistics (n=10, 22%). Furthermore, this main function included subordinated functions such as providing health management guidelines (n=9, 20%) and health information and advice about COVID-19 (n=9, 20%) and sharing the location and helpline number of facilities offering services during this pandemic (n=9, 20%). In addition, some apps (n=5, 11%) provided maps of hotspots or high-risk areas with increased COVID-19 transmission to better inform the public of their travel plans.

**Table 2:** Main functions and subordinate functions of the included apps (N=46).

Main functions and subordinate functions		Apps, n (%)	Apps, %^a^
**Public awareness measures**		19 (41)	—^b^
	News or government measures	12 (26)	7
	Up-to-date statistics	10 (22)	6
	COVID-19 health information	9 (20)	5
	Health management guidelines	9 (20)	5
	COVID-19 related services information	9 (20)	5
	Hotspot/risk area identification	5 (11)	3
**COVID-19 testing**		9 (20)	—
	Obtain COVID-19 test	4 (9)	2
	Report of test results	7 (15)	4
**Quarantine monitoring**		12 (26)	—
	Regular health check	5 (11)	3
	Location tracking	10 (22)	6
**Health monitoring**		32 (70)	—
	Digital contact tracing	11 (24)	7
	Digital check-in	11 (24)	7
	Alert contacts of COVID-19 cases	12 (26)	7
	Report suspected cases/rule infringement	5 (11)	3
	Health code/status generator	7 (15)	4
	Health/travel declaration	7 (15)	4
	Self-symptom assessment	8 (17)	5
**Vaccination**		7 (15)	—
	Vaccination information	4 (9)	2
	Vaccination registration/appointment	3 (7)	2
	Vaccination certificate	4 (9)	2
	Reporting adverse reactions	1 (2)	1
**Health resources**		12 (26)	—
	Virtual medical consultation	4 (9)	2
	Emergency helpline	7 (15)	4
	Accessing medical records	1 (2)	1
	Personal protective equipment distribution	4 (9)	2

^a^% values calculated on the basis of the total functions (n=169).

^b^Not applicable.

Seven (15%) apps supported the function for COVID-19 vaccination. Most of these apps provided information regarding COVID-19 vaccines (n=4, 9%) or issued digital proof-of-vaccination (n=4, 9%) to app users who have completed their vaccine doses. Users could also register and make appointments for COVID-19 vaccination (n=3, 7%) via the app. However, only one of the apps (2%), Taiwan V-watch, allowed users to report vaccination-related adverse reactions.

**Figure 2:**
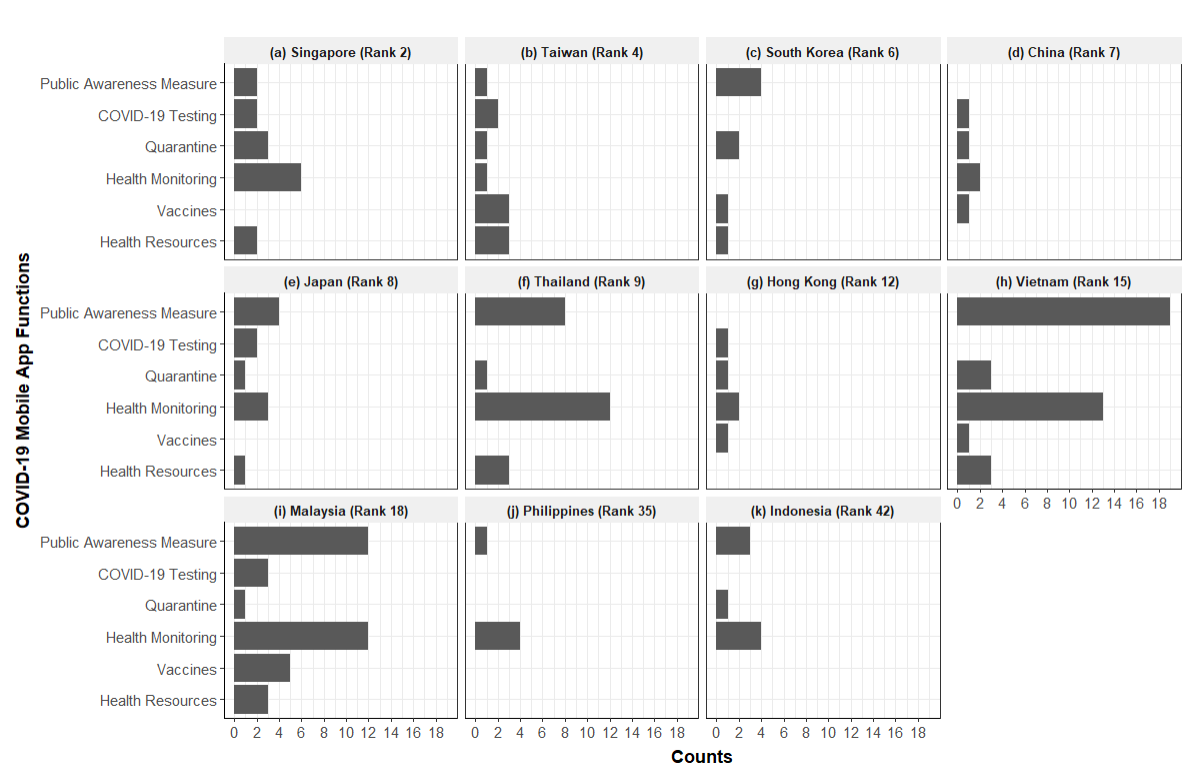
Overview of the key functions of the included government apps.

Figure 2 illustrates the total number of functions served by mobile apps in each government by adding up the number of functions of each app per government. For example, if a government introduced multiple mobile apps having the same functions, the total number of functions will be the sum of each function. Mobile apps in Taiwan and Malaysia had all main functions related to 6 different policy types, and those in Singapore and Japan covered most of the functions except for vaccination. Mobile apps in Thailand, Vietnam, and Malaysia focused on functions for public awareness measures and health monitoring. Among these apps, the MySejahtera app from Malaysia was the most comprehensive app, incorporating public awareness measures, quarantine monitoring, health monitoring, vaccination, and health resources. However, the types of functions served by mobile apps were relatively limited in the Philippines and Indonesia compared to those in the other 9 economies in Bloomberg’s Covid Resilience Ranking.

### Relationship Between Government Measures and the Availability of Mobile Apps

**Figure 3:**
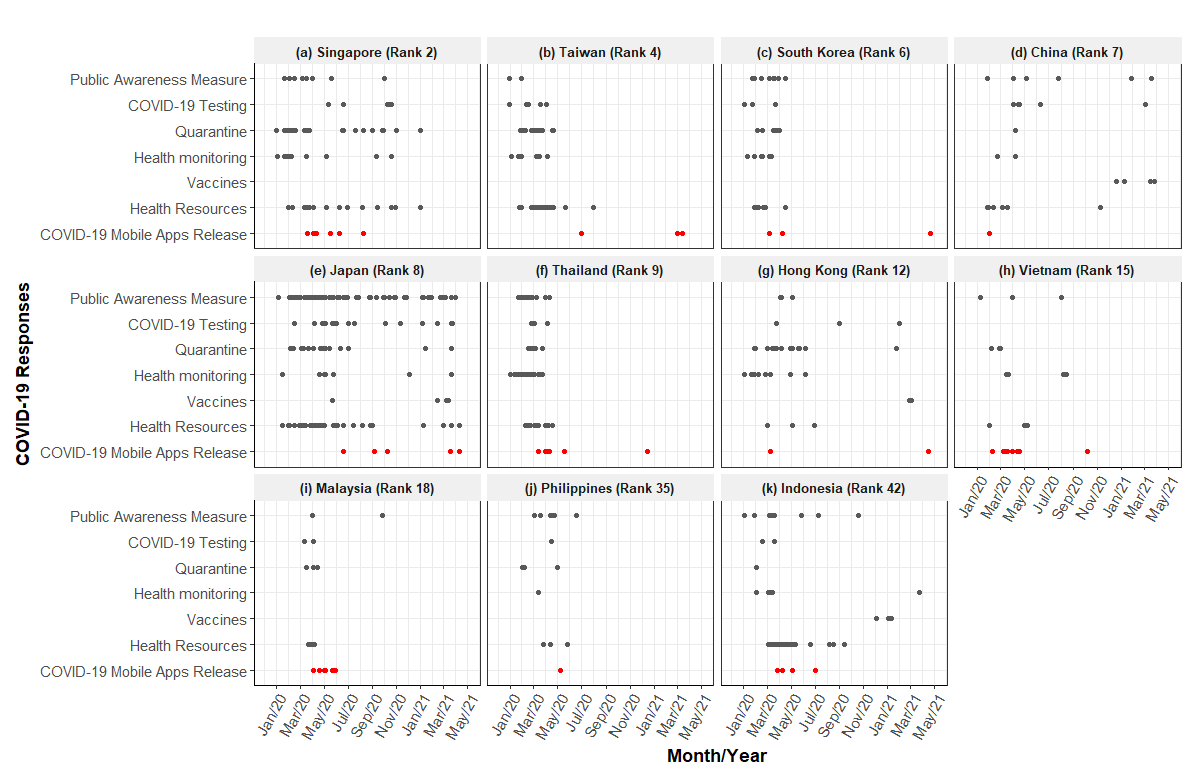
Governments' COVID-19 policy commencement dates and release dates of the included apps.

Figure 3 shows the timeline of the commencement dates of public health policies and the release dates of mobile apps. Each policy type consists of subtypes, and each point indicates the timepoints of when the policies were implemented. We did not examine the details of each policy.

All governments introduced mobile apps to support COVID-19 mitigation policies. There were no noticeable differences among the included governments with respect to the time of introduction of mobile apps. Furthermore, there was no consistency in the introduction of mobile apps and the initiation of certain types of policies across the governments. Eight governments, namely Singapore, South Korea, China (mainland), Thailand, Hong Kong, Vietnam, Malaysia, and Indonesia, launched their first apps between March and April 2020 (Figure 3).

In 2021, Hong Kong, Taiwan, and South Korea released apps to help track COVID-19 vaccination, registrations, and side effects. Some apps such as WeChat (China [mainland]), MySejahtera (Malaysia), Selangkah (Malaysia), and Bluezone (Vietnam) were updated to include vaccination-related functions.

## Discussion

### Principal Findings

This study identified 46 mobile apps developed or supported by 11 governments in East and South-East Asia by using a systematic search method. The most common function was health monitoring. Within the health monitoring function, the most popular function was alerting positive cases, followed by contact tracing and digital check-in. The second-most common function was public awareness measures such as disseminating news or government measures.

Evidence shows that most apps initially focused on disseminating information or monitoring high-risk areas and subsequently had functions for contact tracing [25,33]. As we searched mobile apps cross-sectionally, we did not examine changes in the functions over time. However, most apps in our review had additional functions such as digital check-in, self-assessment of symptoms, virtual medical consultation, COVID-19 testing management, and vaccination-related processes. We noticed that the functions of COVID-19 apps were expanded to cover vaccination-related purposes too. Provision of information and issuance of vaccination certificates were the most frequent functions, followed by vaccination registration or appointment. In other governments, apps not having such functions at the time of our search in May 2021 subsequently integrated the functions in parallel with their nationwide administration of COVID-19 vaccines. In Singapore, test results and vaccination records were added to the pre-existing health information app “HealthHub SG” in February 2021 [34]. In Japan, the COVID-19 vaccination certificate will be available via a QR code using a smartphone in December 2021 [35]. Thus, mobile apps can play an important role in promoting the COVID-19 vaccination programs and increasing their coverage [36].

Since Alanzi [13] reviewed 12 mobile apps in August 2020, we noticed that many mobile apps integrating various functions have emerged. This change might be due to governments’ efforts to address users’ evolving needs and increase data management efficiency by health authorities [37,38]. Furthermore, some governments such as those of Japan, Malaysia, and Vietnam have developed city-level or state-level apps that provided area-specific information, which supported the local health systems. Given the necessity of crisis management at subnational levels, app-based measures can be promising by promoting regional coordination [39].

Most governments in our review required international travelers to use their apps for health declaration and monitoring. Notably, most quarantine monitoring apps were mandatory for people who required quarantine, mainly international travelers. Compulsory implementation of these apps to other settings or populations would not be simple considering national or regional policies regarding data protection and privacy [40]. Indeed, data security and sharing of data with third parties have been the main reason underlying the reluctance to share information in mobile apps [41,42]. Lack of public trust toward authorities is also a significant reason to refuse privacy trade-off [43,44]. Hence, to maximize the effectiveness of the apps, there must be coordinated legal and ethical governance in place to confer protection against invasion of users’ privacy [45].

We examined the timing of the rollout of COVID-19–related mobile apps to assess their relationship with the introduction of other public health measures. All governments included in our review used mobile apps to support the COVID-19 mitigation policies. We found that mobile apps from more successful economies such as Singapore and Malaysia tended to have diverse functions covering various measures. Most apps also first emerged close to the commencement dates of relevant public health policies between March and April 2020. Governments that showed successful performance tended to introduce COVID-19–related apps in the early stages of the pandemic. We did not statistically analyze associations between the timing of introducing apps and epidemiological data. Therefore, further analysis is required.

Although our findings focused on mobile apps, there are various other forms of digital solutions to combat COVID-19. For example, Taiwan did not have a particular mobile app for monitoring quarantine using GPS; however, it initiated the “Entry Quarantine System.” This system was achieved by scanning the QR code directly or clicking on its website. Travelers were required to make a web-based health declaration within 2 days before arriving in Taiwan and complete 14-day quarantine at a government facility, a designated hotel, or at home. Thereafter, the “Electronic Fence system” tracks the locations of individuals during their quarantine period using mobile location data to ensure that travelers do not leave their quarantine location [46]. In China (mainland), AI solutions have been used in lung computed tomographic scans, minimizing time and allowing for early diagnosis of COVID-19 cases [47]. Multifaceted digital approaches were utilized, and although they were not substitutes for traditional health care, their integration complemented and enhanced a functioning health system.

It is difficult to determine which mobile app was the most effective in curtailing COVID-19. As of March 24, 2021, Taiwan and Vietnam recorded 0 deaths per 1 million population, 1 in Thailand, 5 in Singapore, 27 in Hong Kong, 3 in China (mainland), 33 in South Korea, 38 in Malaysia, 70 in Japan, 119 in the Philippines, and 146 in Indonesia [28]. Overall, governments introducing mobile apps covering various forms of public health measures showed fewer deaths per million population. However, other factors such as the health system capacity and resources should be considered. For instance, although Malaysia had the most comprehensive COVID-19 apps in our review, Singapore was the top-performing government with the highest COVID-19 resilience in the Asia Pacific region, having the fastest inoculation program and the lowest positive test rate (Multimedia Appendix 1). Future research could therefore consider other domains of public health to assess the performance of COVID-19–related mobile apps.

Our included apps were purposefully selected from governments, which displayed the most cohesive responses to the pandemic as of March 2021. However, the unprecedented infiltration of the highly transmissible delta variant has wrecked the model of COVID-19 containment success exercised in East and South-East Asia. South-East Asia has emerged as the new virus epicenter; the bottom 5 in the latest Bloomberg’s Covid Resilience Ranking (August 2021) were all South-East Asian economies [48]. Although these economies showed effective resilience by adapting mobile apps in their public health policies, there are still barriers or blind spots that the current mHealth approaches should overcome.

### Practical Implications

This review has several implications for the governments and for public health researchers. Our findings show that governments in East and South-East Asia initiated mobile solutions in the early days of the pandemic, and their COVID-19–related mobile apps were used for various purposes.

Successful performance of mobile apps in both resource-rich and resource-limited settings in this region demonstrated the wide range of applications of these apps and their cost-effectiveness (Multimedia Appendix 3). Although we only compared the timing of the introduction of mobile apps in relation to the commencement dates of other public health policies (Figure 3), we observed how mobile apps are intertwined in the context of public health policies. Governments should consider these mobile solutions in East and South-East Asia to strengthen the current public health system and prepare for subsequent outbreaks.

For public health researchers, there is an enormous potential for such apps, especially in epidemiological research, disease surveillance, and allocation of health resources. Mobile apps can be designed to collect and generate research data to improve our understanding and response to this pandemic.

### Limitations and Recommendations for Future Studies

This study has limitations that are important to acknowledge. It is plausible that some apps may have been missed owing to the restrictive setting of several regional app stores. To overcome this issue, we have scoured other sources of information such as current news articles, media reports, and literature to find additional relevant apps. However, it is still likely that some relevant apps were missed as our search terms may not encompass all the available apps, especially those named in the local languages.

Moreover, we did not collect data on the consumer ratings or user feedback of each app. We also neither examined the popularity nor considered the number of app downloads. Although some evidence suggests that contact-tracing apps should be adopted by at least 60%-70% of the population to impact the outbreak transmission rate, much lower app penetration could still be substantial in breaking transmission chains and preventing infection [49-51]. Nevertheless, given that the number of users determines the utility of mobile apps, our findings may not be generalizable to other countries or populations.

We also did not examine the mobile uptake proportion by people from different socioeconomic backgrounds. There is a need to assess how well these mobile apps were accessible by the most deprived individuals, including older individuals, homeless individuals, immigrants, and rural residents [52-54].

### Conclusions

In conclusion, our findings added knowledge on the COVID-19–related apps used in 11 governments in East and South-East Asia. The most common function was to monitor public health, followed by disseminating information and health education. Most apps deployed GPS technology, followed by Bluetooth and QR scanner technologies. Most countries in this region adopted mobile apps to support COVID-19 mitigation efforts and introduced them close to the relevant policy commencement dates in the early stages of the pandemic. In addition, some governments, which are relatively successful in suppressing COVID-19, tended to have all-in-one mobile apps or other complementary mobile apps. These apps could play pivotal roles in supporting governments’ measures for tracking COVID-19 cases and delivering credible information. Mobile apps catering to the middle-ground strategy of widespread vaccination and reopening of economies can be adopted by the governments to reframe the way of life as we move toward the endemic phase of the COVID-19 pandemic.

## Appendix

Multimedia Appendix 1 Bloomberg’s COVID-19 Resilience Ranking (March 2021).

Multimedia Appendix 2 List of included mobile apps and their associated characteristics.

Multimedia Appendix 3 List of included mobile apps with their associated functions.

## Abbreviations

AI: artificial intelligence
API: application programming interface
ASCN: ASEAN Smart Cities Network
PRISMA-ScR: Preferred Reporting Items for Systematic Reviews and Meta-Analyses Extension for Scoping Reviews
WHO: World Health Organization
