# Supplementary material for: Mobile Apps Leveraged in the COVID-19 Pandemic in East and South-East Asia: Review and Content Analysis
Source: JMIR Mhealth Uhealth. 2021 Nov 11;9(11):e32093. doi: 10.2196/32093 (PMC8589041; doi:10.2196/32093)
Supplement: Multimedia Appendix 1 [file mhealth_v9i11e32093_app1.docx]

**Multimedia Appendix 1.**

| Rank | Country/  Region | Bloomberg’s Covid  Resilience  Score | COVID status | | | | | Quality of life | | | | |
| --- | --- | --- | --- | --- | --- | --- | --- | --- | --- | --- | --- | --- |
|  |  |  | **1-month cases per 100,000** | **1-month fatality rate** | **Total deaths**  **per 1 m** | **Positive test rate** | **Vaccination rate** | **Lockdown severity** | **Community mobility** | **2021 GDP growth forecast** | **Universal Healthcare coverage** | **Human development index** |
| 2 | Singapore | 76.6 | 6 | 0.3% | 5 | 0% | 9.4% | 51 | -11.3% | 5.9% | 92 | 0.94 |
| 4 | Taiwan | 73.5 | 0 | 1.6% | 0 | 0.7% | 0.1% | 25 | -6.8% | 4.3% | 79 | 0.91 |
| 6 | South Korea | 69.7 | 22 | 1.1% | 33 | 1.3% | 0.7% | 58 | -4.6% | 3.4% | 89 | 0.92 |
| 7 | China | 68.4 | 0 | 0% | 3 | 0.1% | 2.7% | 57 | -19.5% | 8.5% | 70 | 0.76 |
| 8 | Japan | 68 | 24 | 4% | 70 | 2.5% | 0.3% | 43 | -13.2% | 2.8% | 96 | 0.92 |
| 9 | Thailand | 67 | 4 | 0.3% | 1 | 0.7% | 0.1% | 47 | -0.2% | 3.5% | 72 | 0.78 |
| 12 | Hong Kong (SAR) | 66.2 | 7 | 1.2% | 27 | 0.1% | 2.6% | 71 | -14.6% | 4.1% | - | 0.95 |
| 15 | Vietnam | 64.2 | 0 | 0% | 0 | 0.1% | 0.1% | 63 | -10.8% | 7.5% | 60 | 0.70 |
| 18 | Malaysia | 61.1 | 135 | 0.4% | 38 | 2.7% | 0.7% | 68 | -22.7% | 5.5% | 67 | 0.81 |
| 35 | Philippines | 51.9 | 102 | 0.8% | 119 | 13.6% | 0.2% | 79 | -29.9% | 7.5% | 55 | 0.72 |
| 42 | Indonesia | 49 | 60 | 2.8% | 146 | 13.7% | 1.6% | 69 | -21.8% | 4.8% | 49 | 0.72 |

**Table S1. Bloomberg’s COVID-19 Resilience Ranking (March 2021)***

*Data from Hong, Chang, & Verley, 2021

**Reference**

Hong, J., Chang, R., & Verley, K. (2021). The best and worst places to be as variants outrace vaccinations. *The Covid Resilience Ranking.* Retrieved from <https://www.bloomberg.com/graphics/covid-resilience-ranking/>
