# Supplementary material for: Mobile Apps Leveraged in the COVID-19 Pandemic in East and South-East Asia: Review and Content Analysis
Source: JMIR Mhealth Uhealth. 2021 Nov 11;9(11):e32093. doi: 10.2196/32093 (PMC8589041; doi:10.2196/32093)
Supplement: Multimedia Appendix 2 [file mhealth_v9i11e32093_app2.docx]

**Multimedia Appendix 2**

**Table S1. List of included mobile apps and their associated characteristics**

| Country/  region of origin | Name of App | Platform | | Date of release/ Date of update | Developer/ owner | Uptake requirement | Target users | Required technology |
| --- | --- | --- | --- | --- | --- | --- | --- | --- |
|  |  | **iOS** | **Android** |  |  |  |  |  |
| China (mainland) | Alipay Health Code | √ | √ | February 2020 | Alipay (Hangzhou) Technology Co., Ltd | Mandatory | General public | GPS, QR scanner |
| China  (mainland) | Wechat Health Code | √ | √ | February 2020 | WeChat | Mandatory | General public | GPS, QR scanner |
| Hong Kong | StayHomeSafe | √ | √ | March 2020 | Government of Hong Kong SAR | Mandatory | Quarantined individuals | GPS, QR scanner |
| Hong Kong | QR Code Verification Scanner | √ | √ | April 2021 | Food and Environmental Hygiene Department | Voluntary | Business owners | QR scanner |
| Hong Kong | LeaveHomeSafe | √ | √ | November 2020 | Government of Hong Kong SAR | Mandatory | General public | GPS, QR scanner |
| Japan | COCOA - COVID-19 Contact | √ | √ | June 2020 | Ministry of Health, Labour and Welfare of Japan | Voluntary | General public | Bluetooth, Exposure Notification API |
| Japan | Chofu City Corona Information | √ | √ | September 2020 | Chofu City | Voluntary | General public | N/A |
| Japan | Overseas Entrants Locator (OEL) | √ | √ | March 2021 | Emergency Assistance Japan Co., Ltd. | Mandatory | Quarantined overseas traveller | GPS |
| Japan | [TeCOT](https://www.meti.go.jp/english/policy/trade_and_invest/tecot/index.html) | √ | √ | April 2021 | Ministry of Economy, Trade and Industry | Voluntary | Outbound travellers | N/A |
| Malaysia | MySejahtera | √ | √ | April 2020 | Government of Malaysia | Mandatory^a^ | General public | GPS, QR scanner |
| Malaysia | Qmunity | √ | √ | April 2020 | Sarawak Digital Economy Corporation Berhad | Voluntary | General public | GPS, QR scanner Facial recognition, |
| Malaysia | COVIDTrace Sarawak | √ | √ | May 2020 | Sarawak State Government | Voluntary | General public | QR scanner Bluetooth, |
| Malaysia | Jejak Johor | √ | √ | May 2020 | Johor State Government | Voluntary | General public | Bluetooth |
| Malaysia | MyTrace | √ | √ | May 2020 | Ministry of Science, Technology and Innovation | Voluntary | General public | Bluetooth |
| Malaysia | SELANGKAH | √ | √ | May 2020 | Selangor State Government | Voluntary | General public | GPS, QR scanner |

| Country/  region of origin | Name of App | Platform | | Date of release/ Date of update | Developer/ owner | Uptake requirement | Target users | Required technology | |  |
| --- | --- | --- | --- | --- | --- | --- | --- | --- | --- | --- |
|  |  | **iOS** | **Android** |  |  |  |  |  |  |  |
| South Korea | Self Quarantine Safety Protection | √ | √ | March 2020 | Ministry of the Interior and Safety | Mandatory | Quarantined overseas travelers | GPS, Bluetooth | |  |
| South Korea | COVID-19 Guidelines Search | √ | √ | April 2020 | Seoul Public Health and Medical Foundation | Voluntary | General public | N/A | |  |
| South Korea | COOV | √ | √ | April 2021 | Korea Disease Control and Prevention Agency and Blockchain Labs | Mandatory | Vaccinated individuals | N/A | |  |
| Singapore | TraceTogether | √ | √ | March 2020 | Ministry of Health and Government Technology Agency (GovTech) | Mandatory | General public | GPS, Bluetooth | |  |
| Singapore | Homer | √ | √ | April 2020 | Government Technology Agency (GovTech) | Mandatory | Quarantined individuals | GPS | |  |
| Singapore | OneService | √ | √ | April 2020 | Ministry of National Development | Voluntary | General public | GPS | |  |
| Singapore | FWMOMCare | √ | √ | May 2020 | Ministry of Manpower (MOM) | Mandatory | Foreign workers | GPS | |  |
| Singapore | SafeEntry QR Scanner | √ | √ | June 2020 | Government Technology Agency (GovTech) | Mandatory | General public | QR scanner | |  |
| Singapore | StayHome@SG | √ | √ | August 2020 | Immigration & Checkpoints Authority | Mandatory | Quarantined individuals | GPS, QR scanner | |  |
| Taiwan | My Health Bank | √ | √ | July 2020 | Taiwan National Health Insurance Administration | Voluntary | General public | GPS | |  |
| Taiwan | Taiwan V-watch  Via LINE app | √ | √ | March 2021 | Taiwan Centers for Disease Control | Voluntary | Vaccinated individuals | N/A | |  |
| Taiwan | Taiwan Social Distance | √ | √ | March 2021 | Taiwan Centers for Disease Control | Voluntary | General public | Bluetooth | |  |
| Thailand | [H4U-COVID19](https://apps.apple.com/th/app/h4u-covid19/id1505777260) | √ | √ | March 2020 | Ministry of Health | Voluntary | General public | N/A | |  |
| Thailand | SydeKick for ThaiFightCOVID | √ | √ | March 2020 | Ministry of Digital Economy and Society and the Ministry of Public Health | Mandatory^b^ | Quarantined overseas travelers | GPS, Bluetooth | |  |
| Thailand | MorChana | √ | √ | April 2020 | Digital Government Development Agency (DCD) | Mandatory^c^ | General public | GPS, QR scanner Bluetooth | |  |
| Thailand | Card2U | √ | √ | April 2020 | Ministry of Digital Economy and Society | Voluntary | General public | GPS | |  |
| Thailand | Thai Chana | √ | √ | May 2020 | Krungthai Bank | Mandatory^c^ | General public | QR scanner | |  |
| Thailand | ThailandPlus | √ | √ | December 2020 | Ministry of Digital Economy and Society and the Prime Minister’s Office | Mandatory | Overseas travelers | | GPS, QR scanner Bluetooth | |

| Country/  region of origin | Name of App | Platform | | Date of release/ Date of update | Developer/ owner | Uptake requirement | Target users | Required technology |
| --- | --- | --- | --- | --- | --- | --- | --- | --- |
|  |  | **iOS** | **Android** |  |  |  |  |  |
| Vietnam | Vietnam Health | √ | √ | February 2020 | Viettel Information and Communications Technology Solutions Center | Voluntary | General public | N/A |
| Vietnam | Vietnam Health Declaration | √ | √ | March 2020 | Vietnam Ministry of Health Office | Mandatory | Domestic and overseas travelers | GPS, QR scanner Bluetooth |
| Vietnam | COVID-19 | √ | √ | March 2020 | International Joint Stock Company (AIC Group) and Electronic Health Administration - Ministry of Health | Voluntary | General public | GPS |
| Vietnam | Hanoi SmartCity | √ | √ | March 2020 | Hanoi Informatics Centre and Hanoi Centre | Mandatory^d^ | General public | GPS |
| Vietnam | NCOVI | √ | √ | March 2020 | Ministry of Information and Communications and Ministry of Health | Voluntary^e^ | General public | GPS, Bluetooth |
| Vietnam | Ncovi Gia Lai | √ | √ | March 2020 | Information Technology and Communication Center of Gia Lai Province | Voluntary | General public | GPS |
| Vietnam | Bluezone-Contact detection | √ | √ | April 2020 | Ministry of Information and Communications and Ministry of Health | Mandatory | General public | Bluetooth |
| Vietnam | CoviTrack | √ | √ | April 2020 | Vietnam Ministry of Health Office | Voluntary | General public | Bluetooth |
| Vietnam | An toàn COVID19 | √ | √ | October 2020 | Ministry of Education and Training | Mandatory | General public | GPS |
| Indonesia | PeduliLindungi (Care amd Protect) | √ | √ | March 2020 | Ministry of Communication and Information Technology | Mandatory^f^ | General public | GPS, Bluetooth |
| Indonesia | 10 Rumah Aman |  | √ | March 2020 | The President’s Staff Office and Ministry of Communication and Information Technology | Voluntary | General public | GPS, Artficial Intelligence |
| Indonesia | eHAC | √ | √ | April 2020 | Ministry of Health (Kemenkes) Republic of Indonesia | Mandatory | Domestic and overseas travelers | GPS |
| Philippines | StaySafe PH | √ | √ | May 2020 | Multisys Technologies Corporation | Mandatory | General public | QR scanner, Bluetooth |

^a^Mandatory only in areas with internet access; ^b^Mandatory only for travelers from high-risk countries; ^c^Mandatory only in red zone provinces with tight coronavirus control; ^d^Mandatory only for suspected and confirmed COVID-19 cases in Hanoi

^e^Voluntary, but people can access the website for health declarations; ^f^Mandatory only for COVID-19 confirmed patients and patients under observation
