# Supplementary material for: Mobile Apps Leveraged in the COVID-19 Pandemic in East and South-East Asia: Review and Content Analysis
Source: JMIR Mhealth Uhealth. 2021 Nov 11;9(11):e32093. doi: 10.2196/32093 (PMC8589041; doi:10.2196/32093)
Supplement: Multimedia Appendix 3 [file mhealth_v9i11e32093_app3.docx]

**Multimedia Appendix 3**

**Table S1: List of included mobile apps and their associated functions**

|  | Public Awareness Measures | | | | | | COVID-19 Testing | | Quarantine Monitoring | | Health Monitoring | | | | | | | Vaccination | | | | Health Resources | | | | |
| --- | --- | --- | --- | --- | --- | --- | --- | --- | --- | --- | --- | --- | --- | --- | --- | --- | --- | --- | --- | --- | --- | --- | --- | --- | --- | --- |
| App Name | News or government measures | Up-to-date statistics | COVID-19 health information | Health management guidelines | COVID-19 related services information | Hotspot/risk area identification | Obtain COVID-19 test | Report of test results | Regular health check | Location tracking | Digital contact tracing | Digital check-in | Alert contacts of COVID-19 cases | Report suspected cases/rule infringement | Health code/status generator | Health/travel declaration | Self symptom assessment | Vaccination information | Vaccination registration/appointment | Vaccination certificate | Reporting adverse reactions | Virtual medical consultation | Emergency helpline | Accessing medical records | | Personal protective equipment (PPE) distribution |
| Alipay Health Code |  |  |  |  |  |  |  |  |  | Y |  |  |  |  | Y | Y |  |  |  |  |  |  |  |  | |  |
| Wechat Health Code |  |  |  |  |  |  |  | Y |  |  |  |  |  |  | Y | Y |  |  |  | Y |  |  |  |  | |  |
| LeaveHomeSafe |  |  |  |  |  |  |  | Y |  |  |  | Y | Y |  |  |  |  |  |  |  |  |  |  |  | |  |
| StayHomeSafe |  |  |  |  |  |  |  |  |  | Y |  |  |  |  |  |  |  |  |  |  |  |  |  |  | |  |
| QR Code Verification Scanner |  |  |  |  |  |  |  |  |  |  |  |  |  |  |  |  |  | Y |  |  |  |  |  |  | |  |
| COCOA - COVID-19 Contact |  |  |  |  |  |  |  | Y |  |  | Y |  | Y |  |  |  |  |  |  |  |  |  |  |  | |  |
| Overseas Entrants Locator (OEL) |  |  |  |  |  |  |  |  |  | Y |  | Y |  |  |  |  |  |  |  |  |  |  |  |  | |  |
| TeCOT |  |  |  |  | Y |  | Y |  |  |  |  |  |  |  |  |  |  |  |  |  |  |  |  |  | | Y |
| Chofu City Corona Information | Y | Y | Y |  |  |  |  |  |  |  |  |  |  |  |  |  |  |  |  |  |  |  |  |  | |  |
| COVIDTrace Sarawak |  | Y |  |  |  |  |  | Y |  |  | Y | Y | Y |  |  |  |  |  |  |  |  |  |  |  | |  |
| Jejak Johor |  |  |  |  |  |  |  |  |  |  | Y |  | Y |  |  |  |  |  |  |  |  |  |  |  | |  |
| MySejahtera | Y | Y | Y | Y | Y | Y |  |  | Y |  |  | Y |  |  | Y | Y | Y | Y | Y | Y |  | Y | Y |  | |  |
| MyTrace |  |  |  |  |  |  |  |  |  |  | Y |  |  |  |  |  |  |  |  |  |  |  |  |  | |  |
| Qmunity |  | Y |  |  |  | Y |  |  |  |  |  | Y |  |  |  |  |  |  |  |  |  |  |  |  | |  |
| SELANGKAH | Y |  |  |  | Y | Y | Y | Y |  |  |  | Y |  |  |  |  |  |  | Y | Y |  |  |  |  | | Y |
| Self Quarantine Safety Protection |  |  |  | Y |  |  |  |  | Y | Y |  |  |  |  |  |  |  |  |  |  |  |  | Y |  | |  |
| COOV |  |  |  |  |  |  |  |  |  |  |  |  |  |  |  |  |  |  |  | Y |  |  |  |  |  | |
| COVID-19 Guidelines Search | Y |  | Y | Y |  |  |  |  |  |  |  |  |  |  |  |  |  |  |  |  |  |  |  |  |  | |
| FWMOMCare | Y |  |  |  | Y |  | Y | Y |  |  |  | Y |  |  |  |  | Y |  |  |  |  | Y |  |  | Y | |
| Homer |  |  |  |  |  |  |  |  | Y | Y |  |  |  |  |  |  |  |  |  |  |  |  |  |  |  | |
| OneService |  |  |  |  |  |  |  |  |  |  |  |  |  | Y |  |  |  |  |  |  |  |  |  |  |  | |
| SafeEntry QR Scanner |  |  |  |  |  |  |  |  |  |  |  | Y |  |  |  |  |  |  |  |  |  |  |  |  |  | |
| StayHome@SG |  |  |  |  |  |  |  |  |  | Y |  |  |  |  |  |  |  |  |  |  |  |  |  |  |  | |
| TraceTogether |  |  |  |  |  |  |  |  |  |  | Y |  | Y |  |  |  |  |  |  |  |  |  |  |  |  | |
| My Health Bank |  |  |  |  | Y |  | Y |  | Y |  |  |  |  |  |  |  |  |  |  |  |  | Y |  | Y | Y | |
| Taiwan V-watch via LINE |  |  |  |  |  |  |  |  |  |  |  |  |  |  |  |  |  | Y | Y |  | Y |  |  |  |  | |
| Taiwan Social Distance |  |  |  |  |  |  |  | Y |  |  |  |  | Y |  |  |  |  |  |  |  |  |  |  |  |  | |
| Mor Chana |  |  |  |  |  |  |  |  |  |  | Y | Y | Y |  | Y |  | Y |  |  |  |  |  |  |  |  | |
| ThailandPlus |  |  |  |  |  |  |  |  |  |  | Y | Y | Y |  | Y |  |  |  |  |  |  |  |  |  |  | |
| Card2U | Y | Y | Y |  | Y | Y |  |  |  |  |  |  |  |  |  |  |  |  |  |  |  |  | Y |  |  | |
| H4U-COVID19 | Y |  |  | Y |  | Y |  |  |  |  |  |  |  |  |  |  | Y |  |  |  |  |  |  |  |  | |
| SydeKick for ThaiFightCOVID |  |  |  |  |  |  |  |  |  | Y |  |  |  |  |  |  |  |  |  |  |  |  | Y |  |  | |
| Thai Chana |  |  |  |  |  |  |  |  |  |  |  | Y | Y |  |  |  |  |  |  |  |  |  | Y |  |  | |
| An toàn COVID19 |  |  |  |  |  |  |  |  |  |  |  |  |  |  | Y |  | Y |  |  |  |  |  |  |  |  | |
| Bluezone-Contact Detection |  |  |  |  |  |  |  |  |  |  | Y |  | Y | Y |  |  |  | Y |  |  |  |  |  |  |  | |
| COVID-19 (Vietnam) | Y | Y | Y | Y | Y |  |  |  |  |  |  |  |  |  |  | Y |  |  |  |  |  | Y |  |  |  | |
| CoviTrack |  |  |  |  |  |  |  |  |  |  | Y |  |  |  |  |  |  |  |  |  |  |  |  |  |  | |
| Hanoi SmartCity | Y |  |  |  |  |  |  |  | Y | Y |  |  |  |  |  |  |  |  |  |  |  |  |  |  |  | |
| NCOVI | Y | Y | Y | Y |  |  |  |  |  | Y |  |  |  | Y |  | Y |  |  |  |  |  |  |  |  |  | |
| Ncovi Gia Lai | Y | Y | Y | Y |  |  |  |  |  |  |  |  |  | Y |  |  |  |  |  |  |  |  | Y |  |  | |
| Vietnam Health | Y | Y | Y | Y | Y |  |  |  |  |  |  |  |  | Y |  |  | Y |  |  |  |  |  | Y |  |  | |
| Vietnam Health Declaration |  |  |  |  |  |  |  |  |  |  |  |  |  |  |  | Y |  |  |  |  |  |  |  |  |  | |
| PeduliLindungi |  |  |  |  |  |  |  |  |  | Y | Y |  | Y |  |  |  |  |  |  |  |  |  |  |  |  | |
| 10 Rumah Aman |  |  | Y | Y | Y |  |  |  |  |  |  |  |  |  |  |  | Y |  |  |  |  |  |  |  |  | |
| eHAC |  |  |  |  |  |  |  |  |  |  |  |  |  |  |  | Y |  |  |  |  |  |  |  |  |  | |
| StaySafe PH |  | Y |  |  |  |  |  |  |  |  | Y |  | Y |  | Y |  | Y |  |  |  |  |  |  |  |  | |
| Total | 12 | 10 | 9 | 9 | 9 | 5 | 4 | 7 | 5 | 10 | 11 | 11 | 12 | 5 | 7 | 7 | 8 | 4 | 3 | 4 | 1 | 4 | 7 | 1 | 4 | |
